# Actinomycosis of the Lower Lip: Report of a Case

**DOI:** 10.1155/2022/6121315

**Published:** 2022-08-02

**Authors:** Manabu Shigeoka, Daisuke Takeda, Masaya Akashi

**Affiliations:** ^1^Division of Pathology, Department of Pathology, Kobe University Graduate School of Medicine, Kobe, Japan; ^2^Division of Oral and Maxillofacial Surgery, Department of Surgery Related, Kobe University Graduate School of Medicine, Kobe, Japan

## Abstract

Actinomycosis is usually a chronic infectious disease caused by *Actinomyces* species, which are anaerobic Gram-positive bacteria that normally colonize the human oral cavity and digestive and urogenital tracts. Although this lesion often occurs on soft tissue around the mandible, cases localized in the lip are very uncommon. We encountered a patient with actinomycosis in the lower lip. A 76-year-old woman with a 5-mm submucosal nodule of the lower lip was referred to our hospital from a dental clinic. The clinical diagnosis was a benign submucosal tumor. Total excision and histological examination were conducted. No oral antibiotic therapy was prescribed. The histological diagnosis was actinomycosis. The postoperative course was uneventful with no signs of recurrence during the 6 months after surgery.

## 1. Introduction

Actinomycosis is a chronic disease caused by the anaerobic Gram-positive bacterium Actinomyces, and *Actinomyces israelii* is known as the most common causative organism [1, 2]. Although cervicofacial actinomycosis is the most frequent clinical form of actinomycosis, this disease is rare nowadays due to its high sensitivity to most antibiotics and the general improvement in oral hygiene [3]. Actinomycosis of the lip is very rare; we identified only six cases in the international literature in a search of the PubMed database using the search terms “actinomycosis” and “lip” [3–8]. In this report, we present an additional case of actinomycosis of the lip and discuss the diagnostics, therapeutics, and pathogenesis.

## 2. Case Presentation

**Figure 1:**
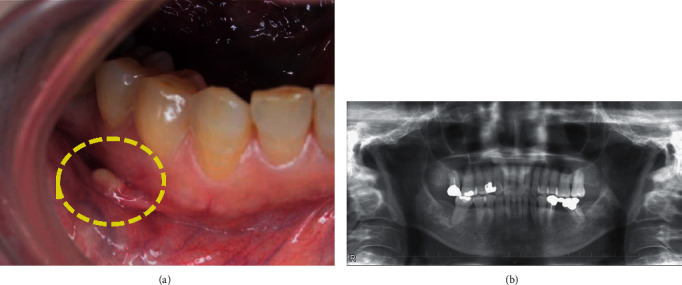
Intraoral clinical findings and imaging findings of the 76-year-old female patient. (a) A whitish-colored submucosal mass covered with normal mucosa was observed on the mucosal aspect of the right lower lip. The *dotted line* indicates the extent of the lesion. This lesion was 5 mm in max. Dia. and was clinically diagnosed as a benign tumor. (b) Dental panoramic radiography provided no significant findings that seemed to be associated with the mass.

A 76-year-old woman with a 2-month history of a submucosal mass on her lower lip was referred to our hospital. She had not received any treatment for the lesion as she had no history of fever, pain, or trauma. An intraoral clinical examination revealed a nonmovable asymptomatic submucosal nodule ~5 mm in diameter on the right side of the lower lip (Figure 1(a)). A whitish color with a creamy tinge was visible through the mucosa. Dental panoramic radiography provided little useful information (Figure 1(b)). The patient had a history of fatty liver, cholecystolithiasis, and spinal canal stenosis.

The clinical diagnosis of a possible benign submucosal tumor was based on the clinical examination findings and the lesion's appearance. A complete excision was performed with the patient under local anesthesia to obtain the histological diagnosis. The surgeon decided not to prescribe antibiotics because the excision was not complex.

**Figure 2:**
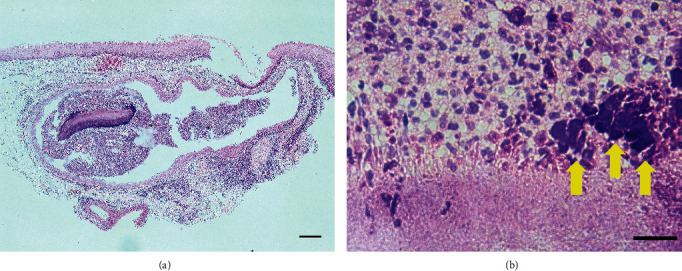
Histological findings. (a) Low-power image. The lesion consisted of an abscess formation with a basophilic bacterial colony contained within a dilated salivary gland duct. Squamous epithelium with little reactive cell atypia covering them was observed. Scale bar: 20 *μ*m. (b) High-power image. The central bacterial colony was characterized by peripheral eosinophilic club-like structures with calcification (*arrows*) and surrounded by inflammatory cells including neutrophils. Scale bar: 200 *μ*m.

Low-power microscopy revealed the bacterial colony within a dilated salivary gland duct in a submucosal area which was covered by non-atypical squamous epithelium (Figure 2(a)). An abscess with numerous inflammatory cells was observed surrounding the bacterial colony. In the high-power view, the colony consisted of a central mass of bacteria surrounded by an eosinophilic club-like structure arranged in radiating rosette pattern with calcification and a dense infiltration of inflammatory cells including neutrophils (Figure 2(b)). The histological diagnosis was actinomycosis. At 6 months after the lesion's excision, the wound had healed well and no symptoms of recurrence were observed.

## 3. Discussion

Several clinical types of actinomycosis have been described, including actinomycosis at a cervicofacial, thoracic, abdominal, or female genitalia site; ~50% of the reported cases occurred in the cervicofacial region [9, 10]. The typical presentation of cervicofacial actinomycosis is the formation of an abscess with a fistula on the skin surface or the oral mucosa and an induration of the soft tissue [2]. Although poor dental hygiene, oral mucosal trauma, and dental intervention have been considered as predisposing conditions for this disease [2], actinomycosis is rare nowadays due to its high sensitivity to most antibiotics and the general improvement in oral hygiene [3]. The majority of cervicofacial sites affected by actinomycosis are the parotids and the mandible [11]. From the anatomical viewpoint, it is speculated that the lip is unlikely to be infected with anaerobic Gram-positive bacteria. In fact, we identified only six cases of actinomycosis of lip in a search of the PubMed database [3–8]. We have presented a patient with actinomycosis of the lip treated by surgical excision and would like to stress three important points learned from this case.

First, an excisional biopsy is practical for the final diagnosis of actinomycosis involving the lip. A histological examination plus bacterial culture has been considered the gold standard for diagnosing cervicofacial actinomycosis [2]. However, in the previous reports of actinomycosis of the lip, five of six cases were not clinically considered a bacterial infection [7, 8]. Consistent with these reports, in the present case, the clinician initially suspected a benign submucosal tumor rather than an infection, and a bacterial culture was not performed. Moreover, it was reported that the results of culture tests may be negative in 50% of actinomycosis cases and suggested that a culture test has no role as a diagnostic aid in tongue actinomycosis [12].

Second, no considerable advantage in prescribing antibiotics may be obtained by treating actinomycosis with a small and limited lesion. Although it is known that actinomycosis can be treated with penicillin [2, 13], our patient was not prescribed antibiotics; because (a) the lesion was not clinically diagnosed as a bacterial infection and (b) the surgeon considered that the risk of postoperative infection was not high. The lesion was treated well by total excision only, and no evidence of recurrence was observed 6 months post-excision. The patient did not show any signs of a lowered immune system related to postoperative infection. It has been proposed that shorter courses of antibiotics are sufficient for small and localized cases with actinomycosis [3]. From the view of antibiotic resistance, it is important that dental clinicians use caution when prescribing antibiotics. The accumulation of further studies regarding the use of antibiotics in cases of small and localized actinomycosis is needed.

Third, we suspect that the lesion in our patient's case was due mainly to the bacterial infection via a duct of a minor salivary gland. Dental manipulation, soft tissue injury, odontogenic infection, poor oral hygiene, diabetes, immunosuppression, malnutrition, irradiation, and neoplasm have each been associated with cervicofacial actinomycosis [12]. Dental panoramic radiography did not reveal any significant findings that would seem to be associated with our patient's lesion, whereas microscopy demonstrated a colony of basophilic club-shaped bacteria arranged in a radiating pattern surrounded within a salivary duct, with normal squamous epithelium covering them. The patient had no history of trauma in the mucosa of her lower lip or at her presentation. Since the histological examination confirmed little reactive epithelial change plus inflammatory cell infiltration, the effects of trauma seem unlikely to apply in her case. The patient also had no history of the systemic conditions described above. However, although she had maintained good oral hygiene, an effect of dry mouth due to aging could not be ruled out. The lips can be mechanically cleaned by saliva, and we speculate that dry mouth caused the bacterial infection in our patient's case. In the dry condition of oral mucosa, bacteria may easily adhere to lip mucosa and invade a minor salivary duct. In fact, a case with the coexistence of actinomycosis and sialolithiasis in a submandibular gland caused by an oral dehydration condition has been reported [10].

In conclusion, a microscopic examination is essential to achieve an accurate diagnosis of actinomycosis involving the lip. Although oral antibiotic therapy may not be necessary for small and localized lesions, further studies are required to clarify this. Our patient's actinomycosis of the lower lip might have been caused by the bacterial infection via a duct of a minor salivary gland.

## Data Availability

The data used to support the findings of this study are available from the corresponding author upon request.
